# Surgery and Interventional Radiology for Benign Bile Duct Strictures

**DOI:** 10.1155/1993/21986

**Published:** 1993

**Authors:** J. E. J. Krige, S. J. Beningfield

**Affiliations:** Departments of Surgery and Radiology University of Cape Town Observatory 7925 Cape Town South Africa


### SURGERY AND INTERVENTIONAL RADIOLOGY FOR BENIGN BILE DUCT STRICTURES

### ABSTRACT

*Schweizer, W.P., Matthews, J.B., Baer, H.U., Nudelmann, L.I., Triller, J., Halter, F., Gertsch, P. and Blumgart, L.H. (1991) Combined surgical and interventional radiological approach for complex benign biliary tract obstruction. British Journal of Surgery; 78: 559–563.*

**In patients with complicated high benign biliary strictures surgical technique alone cannot exclude the possibility of recurrent problems, and hepatic atrophy/hypertrophy, portal hypertension and intrahepatic stones may all complicate surgical management. A multidisciplinary approach to these complex cases, which minimizes the need for repeated surgical interventions, has been pursued. Roux-en-Y hepaticojejunostomy was performed and an extended limb of the jejunum brought to the abdominal wall to allow access for later radiological intervention. Over a 30-month period 58 biliary-enteric anastomoses for benign disease were performed. Seventeen of these 58 patients were managed using the combined approach. Ten of these 17 patients had complex postcholecystectomy strictures and seven had strictures resulting from inflammatory disease, hepatic resection or congenital problems. A new classification of results of management of bile duct strictures is proposed. Seven patients were classified as 'excellent', six 'good', two 'fair' and two 'poor'. Results were obtained at a mean follow-up of 16 months and it seems likely that in some patients major surgical reinterventions were avoided.**

### PAPER DISCUSSION

KEY WORDS: Bile duct stricture, interventional radiology, access loop

Standard surgical techniques of biliary reconstruction offer a high probability of cure for the majority of patients with benign extrahepatic biliary strictures[1–4]. The technical difficulties encountered with high or recurrent strictures are simplified by using a left hepatic duct approach as first described by Hepp and Couinand[5]. However, when a Roux-en-Y jejunal loop is used for the biliary-enteric anastomosis, endoscopic access to the intrahepatic biliary tree is frequently lost. Access is essential in patients with complex recurrent intrahepatic strictures or stones where further problems are anticipated[6]. In the paper under review, Schweizer *et al.* have used percutaneous radiological techniques in selected patients with complex biliary problems as part of a combined approach with surgery using a Roux-en-Y hepaticojejunostomy and creation of an access loop for long-term dilatation. This dual approach is particularly suitable in patients who have had multiple previous operative attempts at repair or portal hypertension, segmental or lobar atrophy and hypertrophy, biliary infection and the presence of intrahepatic strictures and/or calculi which increase the difficulty and risk of surgery[6]. The authors leave a percutaneous silicone tube within the afferent jejunal loop for post-operative radiological manipulation. Once this has been completed, the tube is left *in situ* for a further three months to allow subsequent cholangiography. After removal of the tube, ultrasound was used in two patients to identify the loop for subsequent puncture. In one of these patients surgical exploration was required to find and enter the loop.

The use of a jejunal loop for long-term biliary access has been an important development in the management of complex intrahepatic strictures and stones. This technique allows long-term safe and easy access to the biliary tract while avoiding the morbidity and discomfort of a transhepatic approach and the need for long-term indwelling tubes. The authors have omitted to acknowledge the contribution of Fang and Chou who originally described the afferent loop hepaticojejunostomy to provide unrestricted access to the biliary tree[7]. Later publications by Barker and Winkler[8] and Hutson *et al.*[9] in 1984 reported the use of the access loop in complex cases in Western patients. The initial descriptions used a formal stoma which was unsatisfactory because of bile leakage. Subsequently, a closed loop attached to the peritoneum of the anterior abdominal wall and marked with silver clips has simplified radiological identification[10]. Using this technique in 23 patients we have identified and entered the loop for percutaneous manipulation on 86 of 87 occasions. No problems have been encountered with the blind loop in terms of bacterial overgrowth.

The authors indicate that complete intra-operative clearance of intrahepatic stones or debris was often not possible or was deliberately avoided. We have found that incomplete clearance during surgery of intrahepatic stones especially when associated with strictures, invariably leads to subsequent difficulty in percutaneous retrieval. In particular, segmental branches with multiple stones impacted behind orificial strictures entering at right angles to the main duct are often not visualised or amenable to subsequent percutaneous dilatation and extraction. For this reason we believe it is important to clear as many of the side ducts as possible during the initial surgery. Where significant segmental duct strictures are present, these should be widened by ductoplasty and the impacted stones extracted intra-operatively. In addition, where substantial parenchymal atrophy has occurred, liver resection should be employed as a primary procedure in addition to duct drainage. Most often, left lateral segmental resection is required. When subsegmental ducts are involved and are beyond the reach of conventional operative cholangioscopy, intra-operative radiological manipulation under screening is useful to extract recalcitrant stones.

The technical aspects of the jejunal loop construction are important. The loop should be short to allow direct axial access to the biliary system. We have found a loop length of 12cm to be suitable. A length longer than this creates redundancy and kinking. The loop should generally be sited on the right side of the abdomen except for inferior right duct involvement when a left sided loop is preferable to permit a direct approach. Silver clips on the attached access component of the loop facilitate radiological identification and entry into the loop[10]. Two additional clips on each side of the biliary anastomosis guide catheter advancement and manipulation. Nonabsorbable sutures at the point of attachment to the abdominal wall prevent retraction or dislodgement of the loop during repeated instrumentation. We recommend an interval of three weeks after surgery to allow maturation of the anastomosis before percutaneous manipulation.

The transjejunal retrograde approach facilitates radiological entry to and manipulation within the biliary tree. Cannulation of peripheral ducts from the inferior subhepatic direction via a central duct is technically simpler and the retrograde angle to segmental ducts is less acute than a transhepatic orthograde biliary approach. This direction is critical for selective entry into strictured intrahepatic ducts. It may be necessary to align the afferent jejunal limb with the bile duct using a 9 or 12-F Cook biliary Teflon catheter as a co-axial guide. Larger intrahepatic stones are frequently soft in consistency and can be abraded by rotating a dormia basket alongside the stones within the duct. Once the stones are small enough to pass through the stenosis, they may be extracted using a dormia basket or occlusion balloon catheter or may pass spontaneously after duct irrigation. The use of crushing baskets, ultrasound, laser or intracorporeal electrohydraulic lithotripsy further facilitate stone removal. On completion, a meticulous cholangiographic technique is required to account for each segmental duct. Deliberate catheter probing for unseen side branches is necessary to exclude unsuspected calculi.

The major contribution of the present study that bears emphasis is the prerequisite of a multi-disciplinary approach to high-risk and complex intrahepatic benign biliary strictures in which the magnitude of further surgery and prohibitive morbidity invite alternative strategies. The authors have demonstrated the technical feasibility and short term efficacy of percutaneous access loop instrumentation of the biliary system. However, in view of the tendency of some recurrent strictures to present later and the mean follow-up of 16 months in this study, careful long-term evaluation would be appropriate when endorsing the use of this technique.

J.E.J. Krige
S.J. Beningfield
Departments of Surgery and Radiology
University of Cape Town
Observatory 7925
Cape Town

### REFERENCES

1. Schweizer, W.P., Matthews, J.B., Baer, H.U., Nudelman, L.I., Triller, J., Halter, F., Gertsch, P. and Blumgart, L.H. (1991) Combined surgical and interventional radiological approach for complex benign biliary tract obstruction. *Br. J. Surg.*, **78**, 559–563
2. Terblanche, J., Worthley, C.S., Spencer, R.A.J. and Krige, J.E.J. (1990) High or low hepaticojejunostomy for bile duct strictures *Surgery*, **108**, 828–834
3. Blumgart, L.H., Kelley, C.J. and Benjamin, I.S. (1984) Benign bile duct stricture following cholecystectomy: critical factors in management. *Br. J. Surg.*, **71**, 836–843
4. Pitt, H.A., Miyamoto, T., Parapatis, S.K., Tompkins, R.K. and Longmire, W.P. (1982) Factors influencing outcome in patients with post-operative biliary strictures. *Am. J. Surg.*, **144**, 14–21
5. Hepp, J. and Couinand, C. (1956) L'abord et l'utilisation du canal hepatique gauche dans le separation de la voie biliare principale. *Press Med.*, **64**, 947–948
6. Gibson, R.N., Adam, A., Czerniak, A., Halevy, A., Hadjis, N., Benjamin, I.S., Allison, D.J. and Blumgart, L.H. (1987) Benign biliary strictures: a proposed combined surgical and radiological management. *Aust. NZ J. Surg.*, **57**, 361–368
7. Fang, K. and Chou, T. (1977) Subcutaneous blind loop: a new type of hepaticocholedochojejunostomy for bilateral intrahepatic calculi. *Chinese Med. J.*, **3**, 413–418
8. Barker, E.M. and Winkler, M. (1984) Permanent access hepatico-jejunostomy. *Br. J. Surg.*, **71**, 188–191

9. Hutson, D.G., Russell, E., Schiff, E., Levi, J.J., Jeffers, L. and Zeppa, R. (1984) Balloon dilatation of biliary strictures through a choledochojejuno-cutaneous fistula. *Ann Surg.*, **199**, 637–647
10. Krige, J.E.J., Harries-Jones, E.P., Bornman, P.C. and Terblanche, J. (1987) Modified hepaticojejunostomy for permanent biliary access. *Br. J. Surg.*, **74**, 612–613

